# Large common bile duct stones in high-risk elderly patients: Immediate endoscopic stone removal or elective stone removal? A single-center retrospective study

**DOI:** 10.1186/s12876-023-02976-0

**Published:** 2023-10-05

**Authors:** Ke Meng, Da-ya Zhang, De-xin Chen, Wen-jing Liu, Kai-xuan Fang, Shengxin Chen, Lang Wu, Ming-yang Li

**Affiliations:** 1 Department of Gastroenterology, The First Medical Center, Chinese PLA General Hospital, Fuxing Road, #28, Haidian District, Beijing, 100853 China; 2 Graduate School, Hainan Medical University, Haikou, 571199 China; 3 Graduate School of PLA general hospital, Beijing, 100853 China

**Keywords:** Cholangiopancreatography, Endoscopic retrograde, High-risk, Elderly, Immediate stone removal, Elective stone removal, Efficacy, Safety

## Abstract

**Background and objective:**

For high-risk elderly patients with chronic diseases, endoscopic stone removal for large common bile duct stones is associated with a high risk of adverse events and incomplete stone removal. The aim of this study was to investigate whether the treatment strategy of short-term biliary plastic stent placement followed by elective endoscopic stone removal is more effective and safer than immediate endoscopic stone removal.

**Methods:**

The data of 262 high-risk elderly patients who received endoscopic retrograde cholangiopancreatography (ERCP) for large common bile duct (CBD) stones from 2017 to 2022 were retrospectively analyzed. The patients were divided into group A (immediate stone removal) and group B (stent drainage + elective stone removal). The baseline data of the 2 groups were matched 1:1 by propensity score matching. The stone clearance rate, ERCP procedure time, total hospital stay, and procedure-related adverse events were compared between the matched groups. In group B, stone size before and after stent placement, hospital stay, procedure time and adverse events of two ERCPs were compared.

**Results:**

A total of 57 pairs of patients were successfully matched between the 2 groups. The stone clearance rate in group B was higher than that in group A (89.5% vs. 75.3, *P* = 0.049). The total hospital stay in group B was longer than that in group A (11.86 ± 3.912 d vs. 19.14 ± 3.176 d, *P*<0.001). The total adverse event rate in group A was higher than that in group B (29.8% vs. 12.3%, *P* = 0.005). The incidence of cholangitis/cholecystitis after ERCP was significantly higher in group A than in group B (7.0% vs. 0.9% *P* = 0.029). There was no significant difference in the incidence of post-ERCP pancreatitis, bleeding, pneumonia, and cardio-cerebrovascular events between the 2 groups. There were no perforation cases in either group. After plastic biliary stent placement in group B, the stone size was significantly smaller than before stent placement (1.59 ± 0.544 cm vs. 1.95 ± 0.543 cm, *P* < 0.001), and there was no significant difference in the total adverse event incidence between the two ERCP procedures (18.8% vs. 10.9%, *P* = 0.214).

**Conclusion:**

For high-risk elderly patients with large CBD stones, the treatment strategy involving temporary placement of plastic stent and elective endoscopic stone removal is safer and more effective than immediate stone removal.

## Introduction

Choledocholithiasis is a common biliary disease that can cause abdominal pain, obstructive jaundice, acute cholangitis or pancreatitis and can even be life-threatening. Endoscopic retrograde cholangiopancreatography (ERCP)-related procedures, endoscopic sphincterotomy (EST) and endoscopic papillary balloon dilation (EPBD) have been recognized as a first-line treatment because of less risk and lower mortality than surgery [1]. With the aging of society, there is an increasing number of elderly patients with choledocholithiasis. Although ERCP is minimally invasive and safer than surgery for the treatment of biliary tract disease in elderly patients [2], the risk of ERCP-related adverse events, especially bleeding and cardiovascular and cerebrovascular events, and the risk of mortality are higher in the elderly population than in the younger population [3].

For a common bile duct (CBD) stone ≤ 10 mm in diameter, the complete stone clearance rate for conventional EST and balloon/basket extraction can be as high as 90% [4]. However, for large stones in the CBD (single stone diameter ≥ 1.5 cm or ≥ 3 stones with diameters ≥ 1.0 cm), it is difficult to completely remove the stones by conventional EST or EPBD procedure. And then, endoscopic papillary large balloon dilation (EPLBD), mechanical lithotripsy (ML), or other lithotripsy methods are often required to achieve complete stones removal. However, these methods increase the time and complexity of the ERCP procedure, thereby increasing the risk of complications [5]. Although previous studies have confirmed the efficacy and safety of EST combined with EPLBD for the treatment of large CBD stones in elderly patients [6, 7], there have been few studies on the optimal choice of the treatment strategy for large CBD stones in high-risk elderly patients with chronic diseases (American Society of Anesthesiologists Physical Status classification ≥III).

For difficult CBD stones that cannot be removed endoscopically or for high-risk patients who cannot tolerate surgery and endoscopic stone removal, endoscopic biliary stent placement is a safe and effective treatment option [8, 9]. After a period of biliary plastic stent drainage, CBD stones can decrease in size or become brittle, which is conducive to further endoscopic stone removal [10–12]. The aim of our study was to investigate whether the treatment strategy of temporary stent drainage followed by elective endoscopic stone removal is more effective and safer than immediate endoscopic stone removal for the treatment of large CBD stones in high-risk elderly patients with chronic diseases.

## Materials and methods

### Patient selection and study design

High-risk elderly patients diagnosed with large CBD stones and treated with ERCP between January 2017 and September 2022 in the First Medical Center of PLA General Hospital were retrospectively reviewed. The inclusion criteria were as follows: (1) Age ≥ 65 years; (2) Combined with one or more serious chronic diseases, such as cerebrovascular disease, cardiovascular disease, pulmonary disease, diabetes and chronic renal disease, American Society of Anesthesiologists Physical Status (ASA PS) classification ≥ III; (3) Large CBD stones diagnosed by computed tomography (CT) or magnetic resonance cholangiopancreatography (MRCP) (single stone diameter ≥ 15 mm or ≥ 3 stones with diameters ≥ 10 mm); (4) Unable to tolerate surgery or the patient refused surgery treatment. The exclusion criteria were as follows: (1) The papilla could not be accessed due to anatomical changes caused by gastrointestinal surgery (e.g., Roux-en-Y or Billroth-II reconstruction ); (2) Stones could not be completely removed due to benign or malignant strictures in the distal bile duct; (3) Combined with intrahepatic bile duct stones; (4) Patients had moderate to severe cholangitis according to the criteria outlined in the Tokyo Guidelines (2018) [13]; (5) Patients with malignant tumors or other serious diseases with a life expectancy of less than 6 months.

The patients were divided into two groups, i.e., groups A and B. The patients who received ERCP for immediate stone removal were included in group A (immediate stone removal group). Patients who received a plastic biliary stent placement during the first ERCP and CBD stone removal during the second ERCP were included in group B (stent drainage + elective stone removal). CT, MRCP and ERCP were used to assess the number and diameter of stones. Multiple stones was defined as ≥ 3 stones. Stone diameter was defined as the average of the major and minor diameters of the largest stone.

This study was approved by the Ethics Committee of the First Medical Center of PLA General Hospital (No. S2021-140-01), and all patients signed an informed consent form before ERCP procedure.

### Outcomes

The primary outcome of this study was the complete stone clearance rate, and the secondary outcomes were the incidence of ERCP-related adverse events during hospitalization, ERCP procedure time, and length of hospital stay.

### ERCP procedure

All ERCP procedures were performed under the supervision of an experienced endoscopist with experience in over 1000 ERCP operations. For patients on anticoagulant/antiplatelet medication, anticoagulant/antiplatelet medication was discontinued for an appropriate time prior to ERCP. For the patients who could not stop anticoagulant drugs, low molecular weight heparin was used instead. A standard duodenoscope (TJF-260 V, Olympus Corp., Tokyo, Japan) was used for all ERCP procedures. Wire-guided cannulation was used to intubate the bile duct. When cannulation was unsuccessful by standard methods, the double-wire method for indwelling pancreatic duct wire or the precut technique with a needle knife was used to complete the selective cannulation of CBD. For patients in group A, endoscopists decided to perform EST only or EST combined with EPLBD based on the condition of the papilla. Biliary stone extraction was performed using conventional techniques (basket, balloon, or ML). ML was used when the stones were too large or too hard to remove with a basket or balloon alone. During the procedure, the endoscopist chose the appropriate method for stone extraction based on the specific condition of each patient. For patients in whom stone extraction failed, bile duct drainage was achieved by implantation of a double pigtail plastic stent (10Fr, Cook Endoscopy, Winston-Salem, North Carolina, USA). For patients in group B, a double pigtail plastic stent (10Fr, Cook Endoscopy, Winston-Salem, North Carolina, USA) was placed for bile duct drainage after successful cannulation of the bile duct. The endoscopist decided whether to perform EST before stent placement based on the specific condition of each patient’s papilla. All patients in group B were admitted to the hospital again for a second ERCP 2–10 months later. During the second ERCP procedure, the bile duct stent was first removed under endoscopy. Then, CBD stone removal was performed in the same manner as that for patients in group A. To prevent post-ERCP pancreatitis (PEP), all patients received 100 mg of indomethacin rectally before ERCP. All patients were administered antibiotics after each ERCP, and blood tests were performed on the second postoperative day. The physician decided whether to add blood tests and imaging examination based on the symptoms of each patient and whether there were ERCP-related adverse events.

ERCP-related adverse events were defined as all complications that occurred during hospitalization following ERCP procedure. ERCP-related adverse events included PEP, bleeding, perforation, acute cholangitis/cholecystitis, pneumonia, cardiovascular and cerebrovascular events. The diagnosis and grading of ERCP-related adverse events were based on *A Lexicon for Endoscopic Adverse Events: Report of an ASGE Workshop* by Cotton PB et al. in 2010 [14].

### Statistical methods

Statistical analysis was performed using SPSS 22.0 (IBM Corp., Armonk, New York, USA). To reduce selection bias, a 1:1 propensity score matching (PSM) model was used to match the baseline conditions of the two groups. The caliper value was set to 0.02. All data for continuous variables are presented as the mean ± standard deviation (mean ± SD) or median (interquartile range) [M (*P*_25_, *P*_75_)]. For comparisons between two groups, the Student’s *t* test, one-way ANOVA or nonparametric tests (Mann-Whitney’s *U* test) were employed. Data for categorical variables are expressed as frequencies and percentages. For comparisons between groups, the χ^2^ test or Fisher’s exact test was employed depending on the expected frequency. Two-sided *P* < 0.05 was considered statistically significant.

## Results

**Fig. 1:**
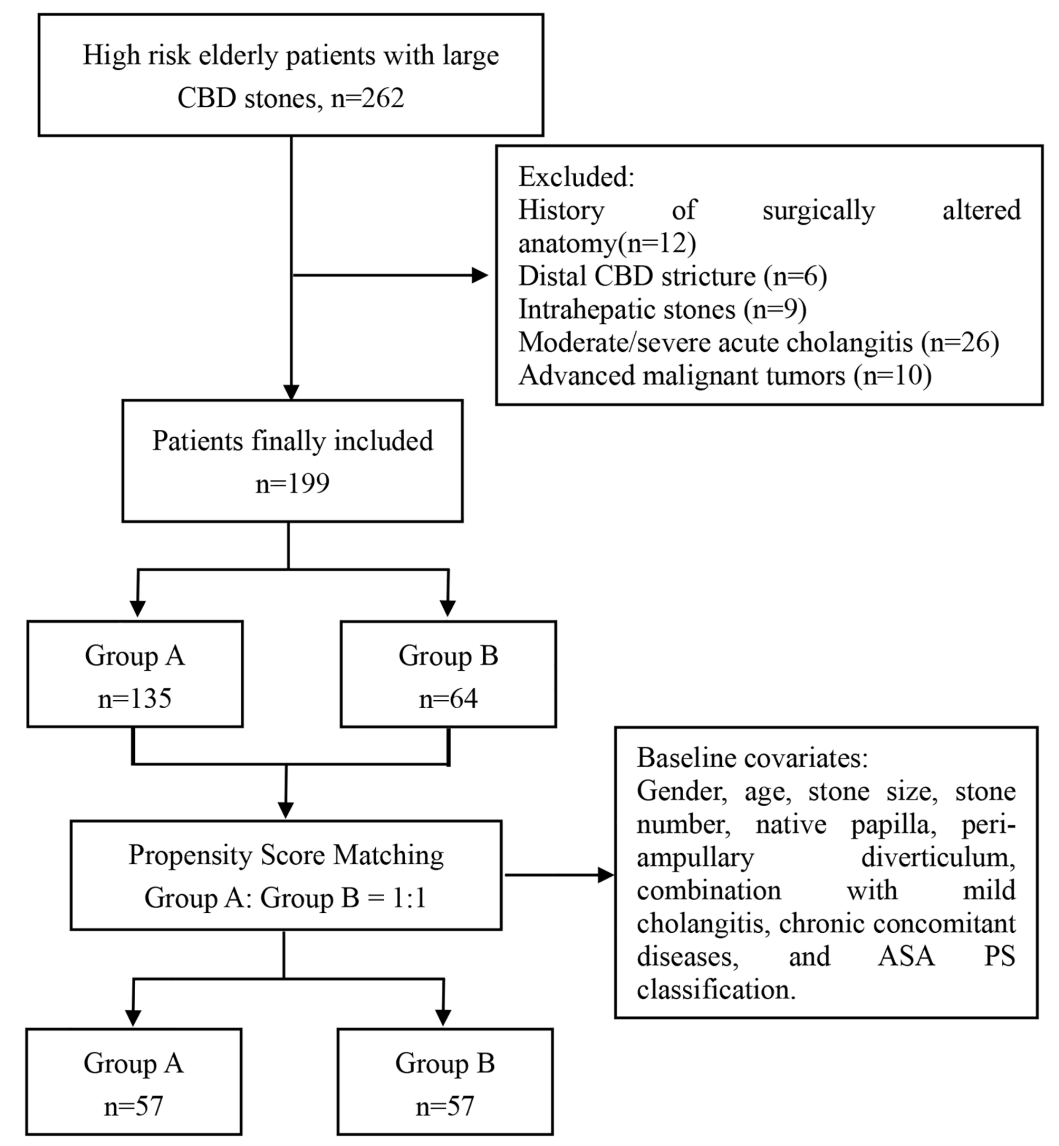
Flowchart of enrolled patients

The data of 262 high-risk elderly patients who underwent ERCP for large CBD stones were reviewed. 12 patients with surgically altered anatomy, 6 patients with strictures in the distal bile duct, 9 patients with intrahepatic bile duct stones, 26 patients with moderate-severe cholangitis and 10 patients with advanced malignant tumors with a life expectancy of less than 6 months were excluded. Finally, 199 patients were included in this study, with 135 patients in group A and 64 patients in group B (Fig. 1).

A comparison of the baseline characteristics of the two groups showed that there were significant differences in age and rate of combination with mild cholangitis between the two groups (Table 1). To reduce the selection bias caused by the differences in baseline characteristics between the two groups, gender, age, stone size, stone number, native papilla, peri-ampullary diverticulum, combination with mild cholangitis, chronic concomitant diseases, and ASA PS classification were used as covariates for PSM analysis (1:1). 114 patients were successfully matched, 57 patients in each group (Fig. 1). The matched groups of baseline characteristics were compared, and the difference was not significant (Table 1).

**Table 1:** Baseline characteristics of patients

Variables	Before PSM			After PSM (1:1)		
	Group A(n = 135)	Group B(n = 64)	*P* values	Group A(n = 57)	Group B(n = 57)	*P* values
Gender(male/female), n(%)	65(48.1%)/70(51.9%)	32(50.0%)/32(50.0%)	0.807	26(45.6%)/31(54.4%)	26(45.6%)/31(54.4%)	1.000
Age (years), median (IQR)	76(70, 81)	79(73, 86)	0.017	78(74, 83)	78(72, 85)	0.836
Diameter of the CBD stone (cm), mean ± SD	1.918 ± 0.5249	1.950 ± 0.5431	0.690	1.905 ± 0.5786	1.923 ± 0.4910	0.862
Number of CBD stones (single/multiple), n (%)	46(34.1%)/89(65.9%)	19(29.7%)/45(70.3%)	0.538	21(36.8%)/36(63.2%)	19(33.3%)/38(66.7%)	0.695
native papilla, n(%)	97(71.9%)	50(78.1%)	0.347	44(77.2%)	43(75.4%)	0.826
Peri-ampullary diverticulum, n(%)	67(49.6%)	33(51.6%)	0.799	32(56.1%)	29(50.9%)	0.573
Acute cholangitis (mild), n(%)	28(20.7%)	26(40.6%)	0.007	20(35.1%)	20(35.1%)	1.000
Chronic concomitant diseases						
Cardiovascular, n(%)	80(59.3%)	39(60.9%)	0.822	42(73.7%)	37(64.9%)	0.310
Respiratory, n(%)	7(5.2%)	2(3.1%)	0.721	2(3.5%)	2(3.5%)	1.000
Cerebrovascular, n(%)	20(14.8%)	11(17.2%)	0.666	12(21.1%)	10(17.5%)	0.635
Diabetes mellitus, n(%)	35(25.9%)	19(29.7%)	0.577	18(31.6%)	14(24.6%)	0.404
Renal, n(%)	8(5.9%)	7(10.9%)	0.252	6(10.5%)	6(10.5%)	1.000
ASA PS Classification (III/IV), n(%)	128(94.8%)/7(5.2%)	57(89.1%)/7(10.9%)	0.149	51(89.5%)/6(10.5%)	53(93.0%)/4(7.0%)	0.508

ERCP procedure: There was no significant difference in the proportion of EST or EST combined with EPLBD between the two groups (*P* = 0.435, *P* = 0.821). The methods of stone extraction between the two groups (balloon, basket, balloon + basket, basket + ML or balloon + basket + ML) was not significantly different (*P* = 0.838). The time of ERCP in group A was longer than the first ERCP in group B (*P* < 0.01), but not significantly different from the second ERCP in group B (*P* = 0.295) (Table 2).

**Table 2:** ERCP related adverse events and outcomes in study patients (n = 114)

	Group A(n = 57)	Group B		*P* values
		First ERCP(n = 57)	Second ERCP(n = 57)	
Complete stone removal, n (%)	43(75.4%)		51(89.5%)	0.049
Procedure time (min), mean ± SD	34.28 ± 15.026^a^	20.96 ± 12.353	37.21 ± 16.943	<0.001
Hospital stay (days), mean ± SD	11.86 ± 3.912	9.77 ± 2.79	9.37 ± 1.665	0.464^b^
		19.14 ± 3.176		<0.001^c^
**Cannulation techniques**, n (%)				
Standard method	47 (82.5%)	44 (77.2%)	57 (100%)	<0.001
Double-wire method	7 (12.3%)	9 (15.8%)	0	
Precut technique	3 (5.3%)	4 (7.0%)	0	
**Therapeutic procedure**, n (%)				
EST	45(78.9%)	39(68.4%)	41(71.9%)	0.435
EST + EPLBD	12(21.1%)		13(22.8%)	0.821
Stone extraction with balloon	2(3.5%)		2(3.5%)	0.838
Stone extraction with basket	34(59.5%)		30(52.6%)	
Stone extraction with balloon + basket	6(10.5%)		10(17.5%)	
Stone extraction with basket + ML	11(19.3%)		12(21.1%)	
Stone extraction with balloon + basket + ML	4(7.0%)		3(5.3%)	
Biliary stent placement	14 (24.6%)	57 (100%)	6 (10.5%)	<0.001
**ERCP-related adverse events, n(%)**	17/57(29.8%)	14/114(12.3%)		0.005
		14/57(24.6%)		0.528
Post-ERCP pancreatitis	8/57(14.0%)	8/114(7.0%)		0.137
Bleeding	1/57(1.8%)	2/114(1.8%)		1.000
Perforation	0	0		NA
Cholangitis/Cholecystitis	4/57(7.0%)	1/114(0.9%)		0.029
Pneumonia	3/57(5.3%)	3/114(2.6%)		0.392
Cardio-cerebrovascular events	1/57(1.8%)	0		0.137

a: Compared with the first ERCP of Group B, *P*<0.001; Compared with the second ERCP of Group B, *P* = 0.295

b: Comparison of two hospital stays in group B, *P* = 0.464;

c: Comparison of the total hospitalization days between group A and group B, *P*<0.001

Outcomes: As shown in Table 2, the complete stone clearance rate in group B was 89.5%, which was higher than that in group A (75.3%, *P* = 0.049). Calculated according to the number of ERCP procedures in each group, the incidence of total ERCP-related adverse events in group A was significantly higher than that in group B [29.8% (17/57) vs. 12.3% (14/114), *P* = 0.005]. Regarding specific types of adverse events, the incidence of cholangitis/cholecystitis after ERCP was significantly higher in group A than in group B (7.0% vs. 0.9%, *P* = 0.029). The incidence of PEP was also higher in group A than in group B (14.0% vs. 7.0%), but the difference was not significant (*P =* 0.137). There were no significant differences in adverse events such as bleeding (*P* = 1.00), pneumonia (*P* = 0.392), and cardio-cerebrovascular events (*P* = 0.137). No occurrences of perforation were reported for each group. Calculated according to the number of patients in each group, there was no significant difference in the incidence of total ERCP-related adverse events between the two groups [29.8% (17/57) vs. 24.6% (14/57), *P* = 0.528]. The hospital stay was significantly longer in group B than in group A (19.14 ± 3.176 d vs. 11.86 ± 3.912 d, *P* < 0.001), and there was no significant difference between the hospital stays for the two ERCP procedures in group B (*P* = 0.464).

**Fig. 2:**
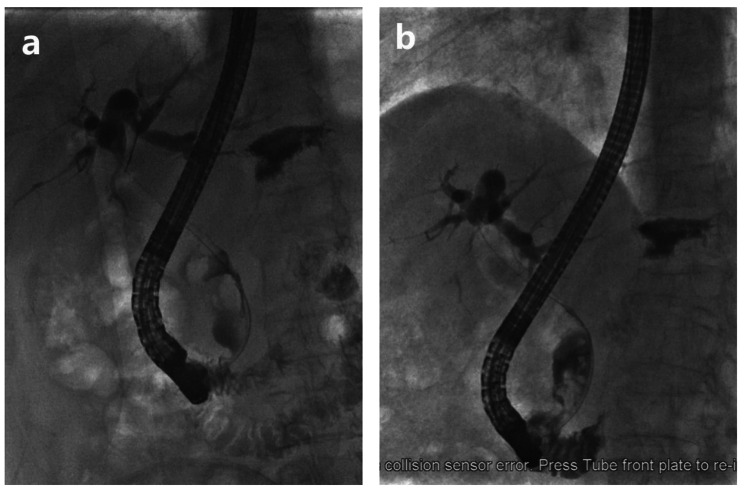
**a**, **b**. ERCP showing the size of CBD stones changed before (**a**) and after (**b**) biliary stent placement

We compared the bile duct stone diameter, ERCP procedure time, adverse event rate and length of hospital stay during two ERCPs for all 64 patients in group B with their own controls (Table 3). The results indicated that after stent drainage, the diameter of CBD stones significantly decreased (1.59 ± 0.544 cm vs. 1.95 ± 0.543 cm, *P* < 0.001) (Fig. 2), and in one patient, the CBD stones completely disappeared during the second ERCP. The ERCP procedure time of the second ERCP was significantly longer than that of the first ERCP (36.70 ± 16.411 min vs. 20.48 ± 11.975 min, P < 0.001). There was no significant difference in the length of hospital stay (10.11 ± 2.885 vs. 9.47 ± 1.681, *P* = 0.132) or the overall ERCP-related adverse event rate (18.8% vs. 10.9%, *P* = 0.214) between the two ERCP procedures. While the incidence of PEP of the second ERCP was significantly lower than that of the first ERCP (3.1% vs. 12.5%, *P* = 0.048). No bile duct stent displacement was found in any patients in group B.

**Table 3:** Comparison of two ERCPs related adverse events and outcomes in Group B (n = 64)

	First ERCP(n = 64)	Second ERCP(n = 64)	*P* values
Diameter of the CBD stone (cm), mean ± SD	1.95 ± 0.543	1.59 ± 0.544	<0.001
Procedure time (min), mean ± SD	20.48 ± 11.975	36.70 ± 16.411	<0.001
Hospital stay (days), mean ± SD	10.11 ± 2.885	9.47 ± 1.681	0.132
**Postprocedure adverse events, n(%)**	12(18.8%)	7(10.9%)	0.214
Post-ERCP pancreatitis	8(12.5%)	2(3.1%)	0.048
Bleeding	1(1.6%)	1(1.6%)	1.000
Perforation	0	0	NA
Cholangitis/Cholecystitis	0	2(3.1%)	0.496
Pneumonia	3(4.7%)	2(3.1%)	0.648
Cardio-cerebrovascular events	0	0	NA

## Discussion

Large CBD stone with a diameter ≥ 1.5 cm, multiple stones, and peri-ampullary diverticulum increase the difficulty of endoscopic stone removal, and the success rate of traditional stone extraction method of EST combined with basket/balloon decreases significantly [9, 15]. Mechanical lithotripsy, EST combined with EPLBD and some novel lithotripsy methods can improve the success rate of complete stone removal. However, the procedure time and complexity of ERCP also increase. For the elderly patients with comorbidities, the risk of procedure related complications and mortality is also increased. Novel lithotripsy techniques such as electrohydraulic lithotripsy (EHL), laser lithotripsy (LL), and extracorporeal shock wave lithotripsy (ESWL) are useful for the successful removal of difficult CBD stones, but these methods are not only time-consuming and expensive [16] but also require special instruments. Many medical institutions are not equipped with these technologies. For CBD stone that is difficult to remove endoscopically, current guidelines recommend the endoscopic placement of stents for bile duct drainage to prevent stone impaction and cholangitis [9, 12]. For patients with a short-expected survival time or who cannot tolerate endoscopic stone removal, biliary stent drainage can be used as an alternative treatment. For difficult CBD stones with failure of initial endoscopic stone removal, short-term biliary stent drainage followed by elective endoscopic stone removal is currently a commonly used treatment strategy. Previous studies have shown that EST combined with EPLBD is safe and effective for large CBD stones removal in elderly patients. However, for high-risk elderly patients combined with chronic diseases and a poor general condition (ASA PS classification ≥ III), few studies have been reported on whether immediate endoscopic stone removal or the short-term placement of bile duct stents for drainage and then elective endoscopic stone removal is safer and more effective.

In this study, we compared the efficacy and safety of temporary bile duct stent placement and elective endoscopic stone removal with those of immediate endoscopic stone removal for the treatment of large CBD stones in high-risk elderly patients. The results showed that the stone clearance rate for patients in the stent drainage + elective stone removal group was higher than that for patients in the immediate stone removal group. In terms of ERCP-related adverse events, the total incidence of adverse events in the stent drainage + elective stone removal group was lower than that in the immediate stone removal group, but the average length of total hospital stay in the former was longer than that in the immediate stone removal group.

EST combined with balloon/basket stone extraction has become the standard treatment for choledocholithiasis, with an overall stone clearance rate of 80-95% [17, 18]. For difficult CBD stones, such as large stones (diameter ≥ 1.5 cm), and multiple stones (number ≥ 3), the total stone clearance rate ranges from 73.7 to 95.6% after short-term biliary stent drainage and elective stone removal [11, 19, 20]. In our study, the success rates for stone extraction were 75.3% and 89.5% in the immediate stone removal group and the stent drainage + elective stone removal group, respectively. The stone clearance rate for stent drainage followed by elective stone removal in this study was lower than that reported in some previous studies, which may be related to the fact that the high-risk elderly population was selected in our study. Due to the poor general condition of these patients, endoscopists tried to avoid prolonged endoscopic stone removal procedures to reduce the risk of adverse events, which affects the overall stone clearance rate. In this study, the stone clearance rate in the stent drainage + elective stone removal group was higher than that in the immediate stone removal group. We hypothesized that this was due to the reduction in the size and hardness of the CBD stones after short-term stent placement. This study showed that after short-term stent placement, the average diameter of CBD stones was significantly smaller than that before stent placement (1.95 cm vs. 1.59 cm). For one patient, no stones were detected at the second ERCP. We speculate that it is the result of spontaneous stone removal after stone fragmentation caused by stent implantation, which has also reported in previous studies [8, 20, 21]. Short-term biliary stents placement can reduce the size and number of CBD stones, but the exact mechanism is not known. It is speculated that the mechanism may be related to the friction between the stent and the stone caused by breathing and intestinal movement, thereby causing fragmentation of the stone. Additionally, the mechanism may also be related to the change in bile composition caused by the reflux of duodenal contents (intestinal fluid or gas) after stent placement [22, 23]. In this study, the biliary stents we used for patients in group B were all double pigtail plastic stents, and no stent displacement or perforation occurred in any patient. Previous studies have shown that double pigtail stents are associated with lower risks of displacement, perforation, and cholangitis [22, 24]. Nonetheless, there are no high-quality studies confirming which stent is most suitable for short-term biliary stent placement.

Regarding ERCP-related adverse events, the total incidence was higher in group A than in group B, calculated according to the number of ERCP procedures in each group. The incidence of cholangitis in group A was 7.0% (4/57), which was significantly higher than that in group B. This finding may be related to the more frequent stone extraction by balloon/basket or mechanical lithotripsy in the bile ducts of patients in group A, resulting in an increase in the contrast dose injection and an increase in the procedure time, all of which increase the risk of biliary infection. Previous studies have shown that elderly patients have a higher risk of cardiovascular events after ERCP. The increasing in ERCP procedure time (> 30 min) significantly increases the risk of myocardial injury in elderly patients [25]. However, in this study, although the ERCP procedure time in group A was significantly longer than that in group B for the first ERCP, there was no significant difference in the incidence of cardiovascular events. Only one patient in group A developed acute coronary syndrome (1.8%, 1/57) after procedure. There were no adverse cardiovascular or cerebrovascular events in group B. This non-significant difference may be due to the small sample size included in our study. When the incidence of ERCP-related adverse events was calculated according to the number of patients in each group, that was also slightly lower in group B than in group A (24.6% vs. 29.8%). Although the difference was not statistically significant (*P* = 0.528), it is reasonable to believe that stent drainage + elective stone removal is safe and does not increase the incidence of ERCP-related adverse events, even if two ERCP procedures are required in each patient. However, it should be noted that 7.8% (5/64) of patients in group B developed biliary infection due to stent dysfunction before the second session of ERCP. Although this was not an ERCP-related adverse event that occurred during a patient’s hospital stay, it does affect the safety of the treatment strategy of elective stone removal. The associated risk factors warrant further study to optimize this treatment strategy.

We also compared the incidence of adverse events of the two ERCP procedures among group B. Although there was no significant difference in the total incidence of ERCP related adverse event between the two ERCP procedures, subgroup analysis showed that the incidence of PEP after the second ERCP was significantly lower than that after the first. This should be due to the fact that after biliary stent placement, the difficulty of bile duct cannulation in the second ERCP is significantly lower than that in the first ERCP, and the time of contact with the papillary and the risk of mistakenly entering the pancreatic duct significantly decreases, thereby reducing the risk of PEP.

There are limitations of this study. First, this was a single-center retrospective study, the sample size was small, may have produced selection bias. Second, there was no further discussion on the types of biliary plastic stents suitable for short-term placement and the optimal indwelling time. Third, since the patients in group B needed to be hospitalized twice and undergo ERCP twice, the medical cost was bound to be higher than that of group A. Due to the policy restrictions of our center, the specific spending amount of the two groups was not available for us when we collected the research data. Therefore, we cannot use detailed figures in the article to compare the medical costs between the two groups. These issues need to be investigated in the future through comprehensive prospective, randomized comparative studies.

In conclusion, the results of our study show that for high-risk elderly patients with large CBD stones, short-term biliary plastic stent placement and elective endoscopic stone removal can reduce the size of CBD stones and the difficulty of stone removal, thereby improving the rate of complete stones clearance and reducing the risk of ERCP-related adverse events, which is important for high-risk elderly patients combined with chronic diseases and a poor general condition.

## Data Availability

The datasets generated and/or analyzed during the current study can be obtained from the corresponding author upon reasonable request.

## Abbreviations

ASA PS: American Society of Anesthesiologists Physical Statu
CBD: Common bile duct
EHL: Electrohydraulic lithotripsy
ESWL: Extracorporeal shock wave lithotripsy
EST: Endoscopic sphincterotomy
EPBD: Endoscopic papillary balloon dilation
EPLBD: Endoscopic papillary large balloon dilation
ERCP: Endoscopic retrograde cholangiopancreatography
IQR: Interquartile range
PEP: Post-ERCP pancreatitis
PSM: Propensity score matching
LL: Laser lithotripsy
ML: Mechanical lithotripsy
SD: Standard Deviation
